# Prediction of Candidate Drugs for Treating Pancreatic Cancer by Using a Combined Approach

**DOI:** 10.1371/journal.pone.0149896

**Published:** 2016-02-24

**Authors:** Yanfen Ma, Jian Hu, Ning Zhang, Xinran Dong, Ying Li, Bo Yang, Weidong Tian, Xiaoqin Wang

**Affiliations:** 1 Department of Clinical Laboratory, The First Affiliated Hospital of Xi’an Jiaotong University, Xi'an, Shaanxi province, P.R. China; 2 Health Science Center of Xi’an Jiaotong University, Xi'an, Shaanxi province, P.R. China; 3 Department of Biostatistics and Computational Biology, School of Life Science, Fudan University, Shanghai, China; 4 SHAANXI Kang Fu Hospital, Xi'an, Shaanxi province, P.R. China; University of South Alabama Mitchell Cancer Institute, UNITED STATES

## Abstract

Pancreatic cancer is the leading cause of death from solid malignancies worldwide. Currently, gemcitabine is the only drug approved for treating pancreatic cancer. Developing new therapeutic drugs for this disease is, therefore, an urgent need. The C-Map project has provided a wealth of gene expression data that can be mined for repositioning drugs, a promising approach to new drug discovery. Typically, a drug is considered potentially useful for treating a disease if the drug-induced differential gene expression profile is negatively correlated with the differentially expressed genes in the target disease. However, many of the potentially useful drugs (PUDs) identified by gene expression profile correlation are likely false positives because, in C-Map, the cultured cell lines to which the drug is applied are not derived from diseased tissues. To solve this problem, we developed a combined approach for predicting candidate drugs for treating pancreatic cancer. We first identified PUDs for pancreatic cancer by using C-Map-based gene expression correlation analyses. We then applied an algorithm (Met-express) to predict key pancreatic cancer (KPC) enzymes involved in pancreatic cancer metabolism. Finally, we selected candidates from the PUDs by requiring that their targets be KPC enzymes or the substrates/products of KPC enzymes. Using this combined approach, we predicted seven candidate drugs for treating pancreatic cancer, three of which are supported by literature evidence, and three were experimentally validated to be inhibitory to pancreatic cancer celllines.

## Introduction

Pancreatic cancer is the leading cause of death from solid malignancies worldwide[1]. The five-year survival rate of patients diagnosed with pancreatic cancer is less than 5%[2]. This poor prognosis can be attributed to an almost symptomless progression, the lack of effective early diagnosis biomarkers, and limitations in the available therapeutic options. Currently, gemcitabine is the only therapeutic drug approved for treating pancreatic cancer, yet the response rate is poor[1]. Developing new therapeutic drugs for pancreatic cancer is, therefore, an urgent need.

A common strategy for drug development is to conduct high-throughput screening against a large pool of molecules, and identify lead compounds that show activity against a given target[3]. However, even though the pool of available small molecules is very large, it is still incomplete and the best compound may not be included. Furthermore, even if a lead compound is identified through high-throughput screening, it may not be successful for clinical use given the complexity of the disease state[4].

The processes for developing a new drug are not only very costly but also time consuming. Recently, drug repositioning (i.e., new therapeutic applications of existing drugs) has provided a promising alternative pathway to new drug discovery[5]. The advantage of using an existing drug is apparent; it has been already approved, making it potentially marketable in a faster and more cost-efficient way by skipping Phase I clinical trials. Numerous approaches have been proposed for drug repositioning[5]. Many of these are based on an analysis of gene expression data[6]. The rationale of gene expression-based drug repositioning is simple: if two drugs elicit similar gene expression patterns, then they might have similar therapeutic effects. Following this rationale, Lamb *et al*. developed the “Connectivity Map (C-Map) project” by generating expression profiles for cultured human cells exposed to thousands of different drugs[7]. The C-Map project has provided a wealth of data that can be mined for identifying new therapeutic uses for existing drugs.

In response to drug treatment, cultured cells may undergo significant alterations at the transcriptome level. However, at the same time, diseased cells (e.g., cancer cells) would also have significant alterations in the expression of a large number of genes[8]. If the alterations in gene expression patterns induced by a drug are negatively correlated to alterations in cancer-specific gene expression patterns, then it is likely that treatment with this drug has the potential to alter gene expression patterns in cancer cells, and consequently cancer cell development. This concept has been explored in numerous studies[9,10]. For example, Kunkel *et al*. identified ursolic acid, that can reduce fasting-induced muscle atrophy, using the approach of negative gene expression correlation[9]. However, this approach is limited in that cultured cells in the C-Map project are not derived from diseased tissues. Thus, the observed negative correlation between drug-induced and disease cell-specific alterations in gene expression may not be biologically meaningful, and the identified drugs may be false positives. Thus, additional measures are needed to select candidate drugs.

Cancer cells undergo significant metabolic alterations and adaptations [8]. For example, in pancreatic cancer cells, there is a significantly increased uptake of glucose that drives uncontrolled cell proliferation[11,12]. The enzyme-coding genes that play important roles in altered cancer cell metabolism are, therefore, potential drug targets [8]. In C-Map, the targets of each drug are known. If the targets are the key pancreatic cancer (KPC) enzyme-coding genes involved in cell metabolism, and if the drug-induced alterations in gene expression patterns are negatively correlated to alterations in pancreatic cancer cells, then it is highly likely that this drug will be of therapeutic use. However, whether the target of a known drug is the gene of a key enzyme, and which enzyme-coding genes are key in pancreatic cancer cell metabolism, are not known.

Recently, we developed an algorithm called Met-express for predicting key enzyme-coding genes in cancer metabolism and have applied this algorithm successfully to lung, liver, and breast cancers[13]. This algorithm involves integrating a cancer gene co-expression network with a metabolic network consisting of enzyme-coding genes. Because Met-express is a general method, it can be applied readily to predict KPC enzyme-coding genes, which can then be used for selecting candidate drugs obtained through gene-expression based analysis. Following this strategy, we predicted seven candidate drugs for treating pancreatic cancer. Three are supported by literature evidence, while the others are worthy of further study.

## Materials and Methods

### Collection and processing of the pancreatic cancer datasets

We applied the following criteria when selecting pancreatic gene expression datasets from the NCBI GEO database[14]. We searched the GEO database with the key words: “human pancreatic cancer”. We required the samples in a pancreatic gene expression dataset be from normal and cancer tissues, instead of from non-tumor tissues, such as peripheral blood mononuclear cells and saliva. We also required the number of nomal and cancer samples be comparable to each other, and the sample size of the gene expression dataset be greater than 30. With these criteria, we obtained three pancreatic gene expression datasets (see Table 1 for GEO accession numbers and descriptions). Any two of the three datasets have more than 90% of common genes.

**Table 1:** Summary of the three pancreatic cancer gene expression datasets.

GEO ID	Sample type	Sample number	Sample information	Reference
GDS4336	Normal/Cancer	45 matching pairs	Pancreatic ductal adenocarcinoma tumor and adjacent non-tumor tissue	[44]
GDS4103	Normal/Cancer	39 matching pairs	ICF cohort: Whole-tissue pancreatic ductal adenocarcinoma	[45]
GDS4102	Normal/Cancer	16 normal, 36 cancer	Pancreatic tumor and normal tissue samples	[46]

The R package for limma[15] was used to normalize gene expression profiles in these datasets. Log2 transformation of expression values was applied if the median expression value in the original dataset was greater than 16. Genes with too many null expression values were removed. To inspect whether the three pancreatic datasets were too similar to each other, we computed a Pearson Correlation Coefficient (PCC) for each pair of the three datasets using the expression profile of the common genes. The PCCs for GDS4336 and GDS4103, GDS4336 and GDS4102, GDS4103 and GDS4102 were 0.702, 0.734, and 0.908, respectively. Thus, genes in these three datasets had correlated but not highly similar expression levels, making it appropriate to use them in this study. Finally, We used limma to identify genes that were differentially expressed (DE) between cancer and normal samples[15]. A DE gene was identified if its adjusted p value (fdr) was below 0.01 and the fold change was greater than 2.

### Collection of C-Map gene rank profiles

Gene rank profiles were downloaded from the C-Map database[7]. A gene rank profile corresponded to a ranked gene differential expression profile for a cultured cell line in response to drug treatment, with the most up- and down-regulated genes ranked lowest and highest, respectively. In total, 6,100 lists of ranked genes were obtained, corresponding to the treatment of five cultured human cell lines with 1,309 drugs.

### Determination of the correlation between DE genes in a pancreatic cancer dataset and the drug-induced ranked gene lists in C-Map

The procedure described in Lamb *et al*.[7] was followed to calculate the correlations between pancreatic cancer DE genes and the drug-induced ranked gene lists from C-Map. Briefly, for a drug-induced gene rank list and the list of DE genes from a pancreatic cancer gene expression dataset, we applied the Kolmogorov–Smirnov test[16] to determine whether these two lists were negatively correlated, i.e., the up-regulated genes in pancreatic cancer were down-regulated in the drug-treated cultured cells (located near the bottom of the drug-induced rank list), and vice versa. The p value from the Kolmogorov–Smirnov test was adjusted by fdr[17]), and the significance threshold for the adjusted p-value was set at 0.1.

### Met-express procedure

Detailed procedures of Met-express are described in our previous publication[13]. Briefly, a cancer gene co-expression network was first constructed for a cancer gene expression dataset by following the procedure described previously[18]. Each network was then partitioned into gene co-expression modules by using Qcut[18]. A receiver operating characteristic curve was plotted for each gene module by using the median gene expression value in each sample to classify cancer versus normal samples, and the area under the receiver operating characteristic curve to determine the cancer-specificity of the gene module. An enzyme-coding metabolic network of 860 genes was constructed as in [13]. Their relationships were established based on enzymatic reactions, i.e., if the product of a metabolic reaction catalyzed by a given enzyme was the substrate of another reaction catalyzed by a second enzyme, then there was a link between the two enzyme-coding genes. Finally, Met-express incorporated both the cancer-specificity information of a co-expression module, and the enrichment of the degree of metabolic links of a gene within the module, to assign an importance score for each enzyme-coding gene. Genes with an importance score above the median were predicted to be key enzyme-coding genes. Met-express was applied to lung, breast, and liver cancers, and its predictions were validated with both literature and experiment evidence[13]. Here, we applied Met-express to predict key enzyme-coding genes in each of three pancreatic cancer datasets.

### Functional enrichment analysis

The GO[19] annotation file was obtained on Nov 23, 2014. The pathway annotations were from MSigDB[20]. Functional enrichment was performed by using Fisher’s test with R. The p value was adjusted by fdr and the significance threshold was set at 0.1.

### Cell culture and MTT assay

“Human pancreatic cancer cell lines (PANC-1, BxPC-3) were obtained from ATCC (American Type Culture Collection, Manassas, VA) and maintained as ATCC suggested. The cell culture media contained 10% FBS and 1% penicillin/streptomycin. The effects of the test compounds on the cell viability were determined using the MTT (3-(4,5)-dimethylthiahiazo (-z-y1)-3,5-di-phenytetrazoliumromide) assay. Briefly, the exponential cells were exposed to Trypsin (Amresco, 0457) to dissociate adherent cells, and diluted to 1–10×104/mL cells suspension. The cells suspension was grown in 96-well plates at 1×104 cells per well at 37°C with 5% carbon dioxide for 24 hr. The cells were then treated with the compound Biotin, Finasteride and Progesterone or DMSO vehicle as control for 48hr at the corresponding concentration, 1×10–5, 10–4, 10–3, 10–2, 10–1 and 1 mg/mL, with three repeats in each concentration. After that 20 μL of MTT solution (5 mg/mL; AMRESCO Inc, 0793-1G) was added to each well and incubated for 4 hr at 37°C to react with active cells forming formazan crystals. After removing the supernatant, the formazan crystals were dissolved in 150 μL of DMSO and the absorbance (OD) at 570 nm was recorded using the microplate reader (Rayto, Rt2100c). The drug inhibition on cells was then calculated as: 1 –(OD_compound_−OD_blank_)/(OD_DMSO_−OD_blank_).

## Results

### Identification of drugs whose induced-gene rank lists in C-Map negatively correlated with DE genes in pancreatic cancer

**Fig 1:**
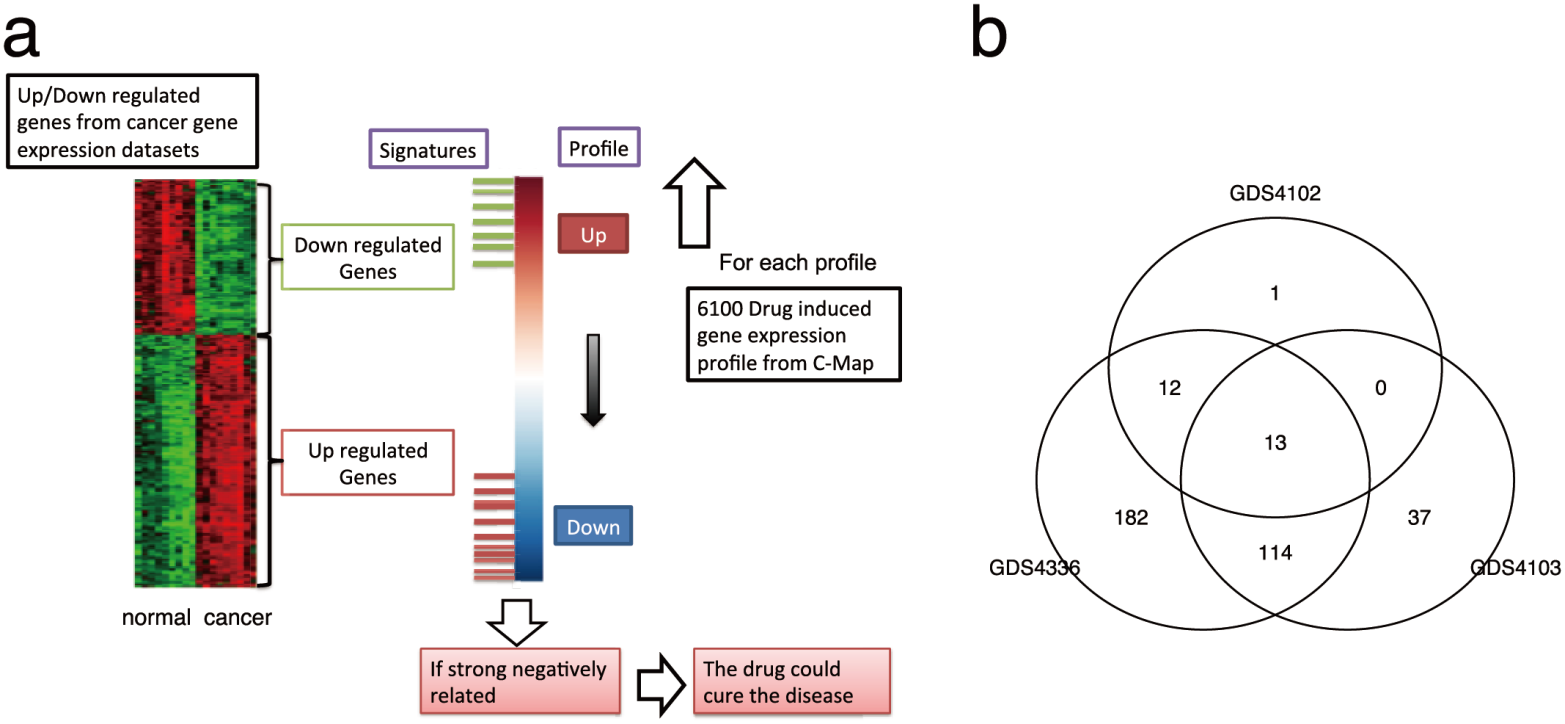
Identification of potentially useful drugs (PUDs) for treating pancreatic cancer using gene expression-based correlation analyses. A. Workflow. B. Venn Diagram for the PUDs identified for each of the three pancreatic cancer datasets.

The C-Map database provides thousands of ranked gene lists for cultured cell lines in response to drug treatments. In each list, genes are ordered according to their DE values between drug-treated and control cell lines. Up- and down-regulated genes are ranked at the top and bottom of the list, respectively. Once a list of DE genes in a pancreatic cancer dataset has been identified, the correlation between the DE genes and each of the drug-induced gene rank lists (Fig 1A) can be calculated. If the correlation is negative and statistically significant, then it is likely that this drug has the potential to reverse the gene expression pattern of pancreatic cancer cells, and consequently may be useful for treating this disease.

From the C-Map database, 6,100 ranked gene lists were obtained, corresponding to the treatment of five cultured human cell lines with 1,309 drugs. We also collected three pancreatic gene expression datasets, and identified the respective DE genes in each dataset. We then computed the correlation between the DE genes in each dataset and each of the drug-induced gene rank lists. These correlations were used to identify all drugs whose induced gene rank lists showed a statistically significant negative correlation with the DE genes (adjusted p < 0.1). Because each drug may have been used to treat multiple cell lines, a drug was considered to be potentially useful in pancreatic cancer if the induced gene rank lists in more than half of the cultured cells showed a significant negative correlation with the DE genes of that cancer dataset. In total, 359 drugs were found to be significant to at least one of the pancreatic cancer datasets. Out of these drugs, 139 and 13 were significant to at least two and all three pancreatic cancer datasets, respectively (Fig 1B). Both numbers were significant compared to the case in which we randomly selected the same number of drugs significant to a pancreatic cancer dataset, and then inspected how many of them were significant to two or all three significant datasets (randomization were repeated 1000 times, and both p-values< 0.001). The 126 drugs that were significant in at least two pancreatic cancer datasets were considered potentially useful drugs (PUDs) for treating pancreatic cancer.

### Application of Met-express to predict key enzyme-coding genes in pancreatic cancer cells

The cultured human cell lines treated with drugs in the C-Map database were not derived from pancreatic tissues. Consequently, it was difficult to interpret the mechanism of the negative correlations between the drug-induced gene rank lists and the DE genes in pancreatic cancers. Thus, many PUDs may be false positives. To solve this problem, we applied Met-express[13] to predict the KPC enzyme-coding genes. Our rationale is that, if the target of a PUD is a KPC enzyme, then the chances that this drug would alter the gene expression profiles of pancreatic cancer are increased significantly. In addition, if a PUD is the substrate or product of an enzymatic reaction catalyzed by a KPC enzyme, then the chances that it would perturb cancer metabolism are also increased. Consequently, PUDs with either of the above two properties are considered candidate drugs for treating pancreatic cancer.

**Fig 2:**
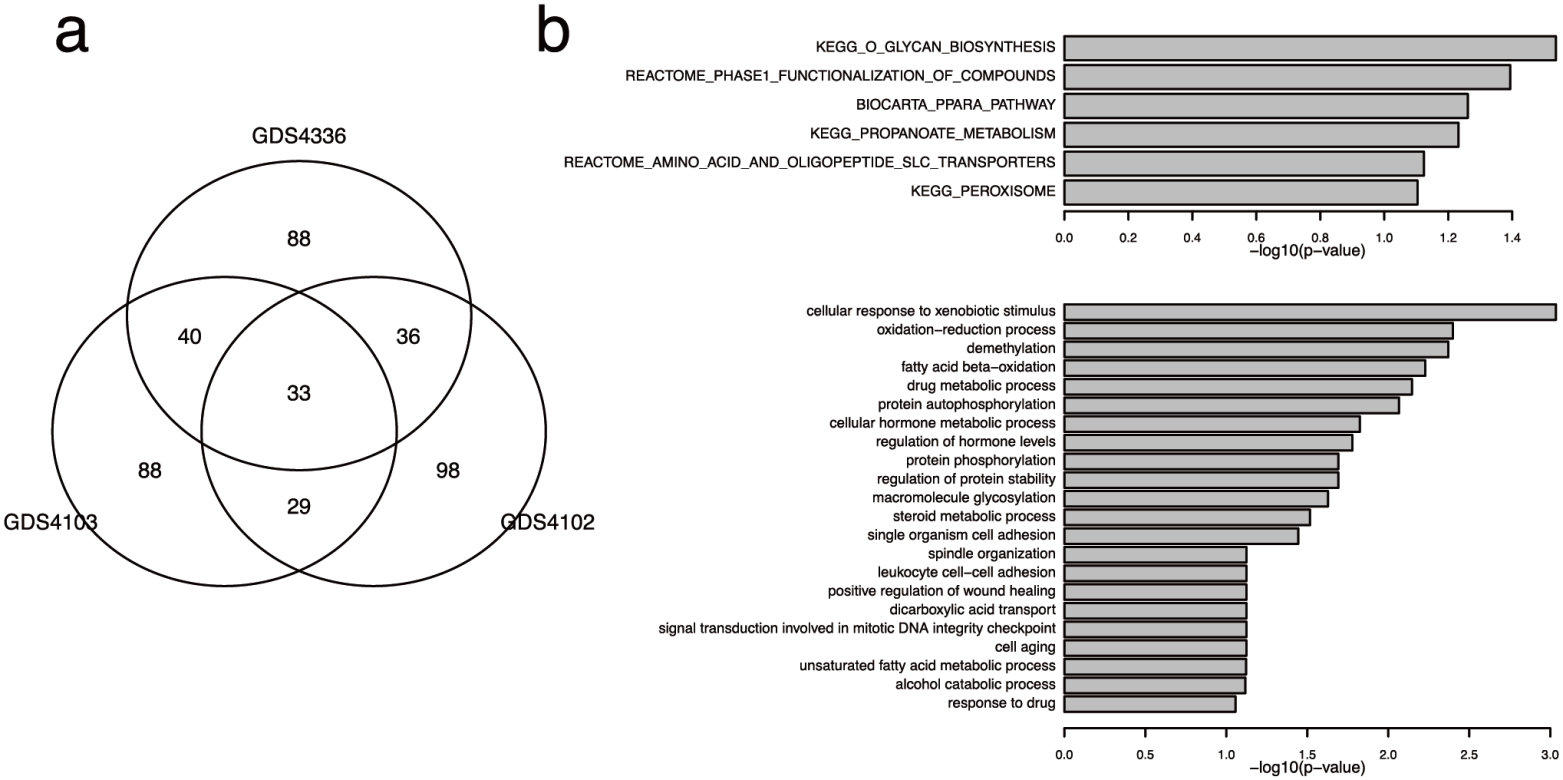
Summary of the predicted key pancreatic cancer (KPC) enzymes. A. Venn diagram for the predicted key enzyme-coding genes for each of the three pancreatic cancer datasets. B. The enriched pathways and biological processes for the KPC enzymes predicted in all three cancer datasets.

Met-express was applied to each of the three pancreatic cancer datasets. A total of 33 key enzyme coding genes predicted in all three datasets were considered KPC enzymes (Fig 2A, S1 Table). Before using these KPC enzymes to select candidate drugs from PUDs, we validated the predictions by both functional enrichment analyses and literature reviews. The 33 KPC enzymes were enriched with 66 GO biological process terms and 11 MSigDB pathways. The top enriched MSigDB pathways were related to O-glycan biosynthesis pathways, while the top enriched GO terms were functions related to xenobiotic stimuli, fatty acid beta-oxidation, drug metabolic processes, and more (Fig 2B, S2 Table).

Abnormal expression of glycoproteins on the surface of cancer cells has been reported [21]. Increased expression of enzymes in glycan-related biological processes promoted cell detachment and invasion, and enzymes in those pathways were proposed to be novel targets for cancer treatment[22]. In addition, aerobic glycolysis was frequently activated in pancreatic tumor cells[23]. The reduced activity of enzymes involved in xenobiotic metabolism is associated with the susceptibility of the pancreas to carcinogenesis[24]. Several risk factors, such as diabetes and high-fat foods, are also related to pancreatic cancer[25,26]. These literature reports demonstrate that the predicted KPC enzymes are strongly related to the development of pancreatic cancer.

### Using the predicted KPC enzymes to select candidate drugs for treating pancreatic cancer

Using the predicted KPC enzymes identified through Met-express, we considered a PUD as a candidate drug for pancreatic cancer if: 1) one of its targets was a predicted KPC enzyme, or 2) it was either the substrate or product of the enzymatic reaction catalyzed by a predicted KPC enzyme. The targets of each PUD, and the compounds corresponding to each PUD, were obtained from the DrugBank database[27]. Four PUDs were found whose targets were the predicted KPC enzyme, and three were found that were the substrate or product of the enzymatic reactions catalyzed by six KPC enzymes (Table 2). These seven PUDs were considered as candidate drugs for treating pancreatic cancer.

**Table 2:** Predicted candidate drugs for treating pancreatic cancer.

Relationships	DrugBank ID (name)	Indication*	Compound ID (name)	The relevant KPC-enzyme
Drug target enzyme	DB08313 (nocodazole)	Not available	-	HPGDS
	DB00121 (Biotin)	Nutritional supplementation, treating dietary shortage or imbalance.	-	MCCC1
	DB01176 (Cyclizine)	For prevention and treatment of nausea, vomiting, and dizziness.	-	SULT1E1
	DB01216 (Finasteride)	For the treatment of symptomatic benign prostatic hyperplasia (BPH) in men and etc.	-	AKR1D1
Compound target enzyme	DB00396 (Progesterone)	For treatment for infertile women with progesterone deficiency and etc.	C00410 (Progesterone)	AKR1D1
	DB04557 (Arachidonic Acid)	Not available	C00218 (Methylamine)	CYP2J2, CYP2C18, CYP2C9
	DB00755 (Tretinoin)	For the induction of remission in patients with acute promyelocytic leukemia (APL) and etc.	C00777 (Retinoate)	CYP2C18, CYP2C9, CYP3A7, CYP3A5

* Indication is obtained from DrugBank.

**Fig 3:**
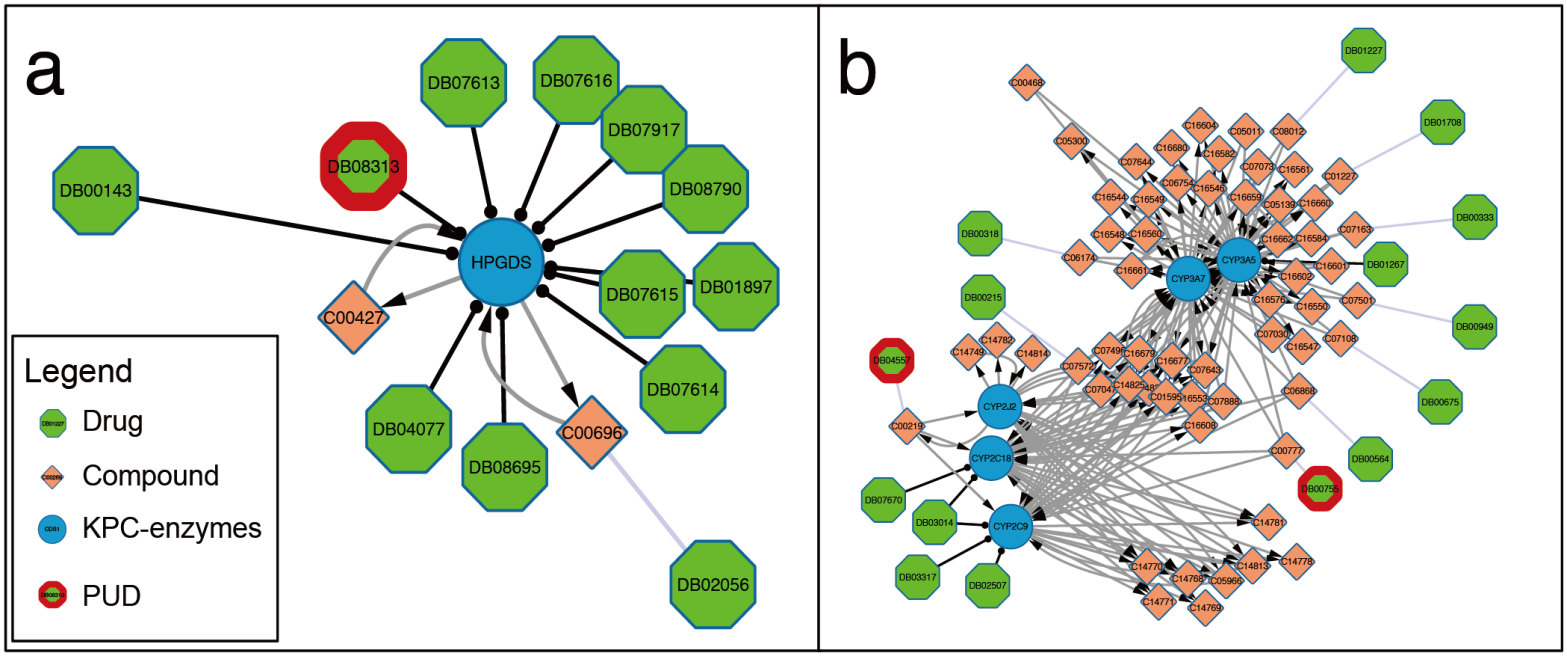
Network structure illustrating the relationships between the selected candidate drugs for treating pancreatic cancer and the relevant KPC enzymes. The examples of selected drugs are: A. nocodazole; B. tretinoin and retinoate.

Nocodazole (DrugBank ID: DB08313) has not been used for treating pancreatic cancer but was one of the candidate drugs whose targets included predicted KPC enzymes. It is an anti-neoplastic agent that interferes with the polymerization of microtubules[28]. Nocodazole has been reported that it has some reversible effects dealing with multidrug resistance in pancreatic cancer cell[29]. In addition, as one of the mitotic spindle-disrupting agents, Nocodazole leads to a mitotic arrest and defects a majority of pancreatic cancers with aneuploidy caused by chromosomal instability[30,31]. According to DrugBank, Nocodazole has only one target that is a predicted KPC enzyme, hematopoietic prostaglandin D synthase (HPGDS) (Fig 3A). HPGDS is a member of the sigma class glutathione-S-transferase family, which catalyzes the conversion of PGH2 to PGD2. This enzyme is involved in the integrated pancreatic cancer pathway (Biosystem ID: 711360).

Tretinoin and Arachidonic acid are two examples of candidate drugs that are substrates or products of the predicted KPC enzymes. Tretinoin (DrugBank ID: DB00755), also called retinoate, is a substrate of the enzymatic reactions catalyzed by three KPC enzymes: CYP2C18, CYP3A7, and CYP3A5 (Fig 3B). Arachidonic acid (DrugBank ID: DB04557) is the substrate for two KPC enzymes, CYP2C9 and CYP2C18, and also the product of one KPC enzyme, CYP2J2. Interestingly, the five KPC enzymes related to DB00755 and DB04557 are all involved in linoleic acid metabolism and biological oxidations (Fig 3B).

Tretinoin is the carboxylic acid form of vitamin A and has been used for treating acne vulgaris and keratosis pilaris. Recently, Guo*et al*. showed that treating the human pancreatic cancer cell line, PANC-1, with tretinoin could dose-dependently inhibit the growth of these cells[32]. This suggested that the antitumor effects of Tretinoin were associated with G2/M phase arrest. Other researchers have reported that Arachidonic acid can sinificantly reduced the growth rate of some pancreatic cancer cell lines such as PANC-1, MIA PaCa-2 and CFPAC[33].

There are also some similar compounds to PUDs that have been used or reported for pancreatic cancer treatment. For example, Vincristine and Demecolcine are similar compounds to Nocodazole, which act on microtubulesand interfere with cell cycle[34,35]. Vincristine, along with 5-fluorouracil-based combination regimens, have been shown to be effective in pancreatic cancer patients’ survival extension compared with no chemotherapy treatment[36]. Demecolcinearrests the early mitosis in both pancreactic cell lines PANC-1 and BxPC-3 cells[37]. Also, Retinol (Vitamin A) and Ergocalciferol (Vitamin D_2_) are similar compounds with Tretinoin, which have been suggested that intake of Vitamin D, Retinol and other multivitamin supplement may reduce the risk of pancreatic cancer[38].

To validate the identified PUDs in cancer cell lines, we managed to search drug response data in some database with cancer cells. The US National Cancer Institute 60 human cancer cell lines(NCI-60) which can be accessed by CellMiner[39] (http://discover.nci.nih.gov/cellminer)includes 60 cancer cell lines represent nine human cancers: breast, central nervous system, colon, kidney, leukemia, lung, melanoma, ovary, and prostate, with allowed rapid data of transcripts for 22,379 genes and 360 microRNAs along with activity reports for 20,503 chemical compounds including 102 drugs approved by the U.S. Food and Drug Administration (FDA)[40]. There were no data on pancreatic cancer cell lines in NCI-60, making it impossible to directly inspect the drug-responsive data of our predicted PUDs on pancreatic cancer cell lines. However, the drug responsive data of PUDs on other cancer cell lines in NCI-60 could indirectly help us to infer their potential usefulness on pancreatic cancer cell lines.

Among seven PUDs we have identified, three of them can be found in the drug zscore patterns of NCI-60, including Nocodazole (NSC #238159), Progesterone (NSC #9704) and Tretinoin (NSC #122758). It has been reported that compounds activity -log_10_GI50(50% growth-inhibitory levels) larger than 6 are considered active[41]. For up to 60 cancer cell lines, we managed to query whether our PUDs is active to them. Using CellMiner NCI-60 Analysis Tool, we searched the drug activity patterns across 60 cell lines and found that for Nocodazole and Tretinoin, they have 55 and 2 cell lines whose activity larger than 6, respectively. The largest activity for Progesterone is 5.94. This has indicated that the three PUDs found in NCI-60 also have inhibition effects to other cancer cells more or less.

### Experimental validation for drug prediction

Besides Nocodazole, Tretinoin and Arachidonic Acid with publication evidence, three of the other four PUDs: Biotin, Finasteride and Progesterone were selected for further experimental validation. We conducted MTT assay on the pancreatic cancer cell lines PANC-1 and BxPC-3 to estimate the cancer cell viability treated with the drugs. Compared with PANC-1, BxPC-3 was cultured from a patient with later-stage tumor who died 6 months later. As both from pancreatic duct, PANC-1 tends to display fewer ductal features than BxPC-3[42]. For cell migration, PANC-1 is better than BxPC-3. While BxPC-3 showed a high angiogenic potential, and PANC-1 showed variable results[42].

**Fig 4:**
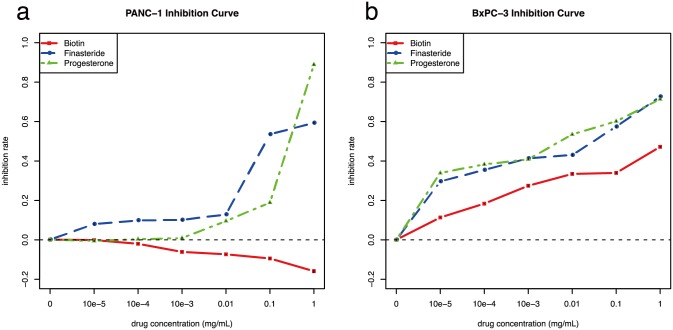
Drug inhibion rate on the Panc-1 pancreatic cancer cell line. A. Pancreatic cell line PANC-1. B. Pancreatic cell line BxPC-3. X and Y coordinates denote concetration and inhibition rate of the three drugs, Biotin, Finasteride and Progesterone. Short line segments on the points denote the variation of three repeats.

Results (Fig 4, S3 Table) showed that when the concentration was increased to 0.1 mg/mL, three of the two drugs,Finasteride and Progesterone, have evident cancer cell inhibition impacts on PANC-1, while inverse effects for Biotin. As for advanced cancer cell line BxPC-3, the lethality of all three drugs became severe with the concentration incereasing, similar for Finasteride and Progesterone, and weaker for Biotin. BxPC-3 showed a more sensitivity to drug treatments than PANC-1, which indicated that these drugs may have a more influence on thetumor angiogenesis.

We further went through the drug impacts on other cancers and found that Progesterone (FDA approved) is responsible for embryo implantation, pregnancy maintenance, and the development of mammary tissue for milk production, which is secreted by the corpus luteum and the placenta. It hasbeen reported as a protective factor for ovarian and endometrial cancers with little known of the mechanism[43]. In the NCI-60 drug pattern database (http://discover.nci.nih.gov/cellminer/), Progesterone is particularly sensitive to the lung cancer cell line LC: EKVX, leukemia cell line LE: RPMI_8226, breast cancer cell-line BR: HS578T, for 5.94, 5.49, and 5.48 as compounds activity -log10GI50, respectively.

Overall, evidence in the literature and experiment suggests that at least five of our predicted candidate drugs (Nocodazole, Tretinoin, Arachidonic Acid, Progesterone, Finasteride) are likely to be useful for treating pancreatic cancer, while the remaining two drugs are worthy of further study.

## Discussion

In the current study, we describe the development of a novel combined approach for predicting candidate drugs for treating pancreatic cancer. In contrast to previous drug repositioning approaches that focused on exploiting correlations between gene expression and drug-induced gene rank lists in C-Map, and the DE genes under disease conditions for predicting PUDs, our approach identified PUDs for pancreatic cancer through both gene expression correlation analyses and an added measure for selecting candidate drugs from the list of PUDs. This latter selection method used a recently developed algorithm, Met-express, to predict the KPC enzyme-coding genes and then used the KPC enzymes for selecting candidate drugs. The combined approach identified seven candidate drugs for treating pancreatic cancer, among which three were supported by literature evidence and another two were supported by experiment. This supported the usefulness of this approach for identifying candidate drugs for treating pancreatic cancer.

The use of KPC enzymes predicted by Met-express is a key component of our approach. According to the algorithm design of Met-express, the predicted key enzyme-coding genes should share the following two properties: 1) their expression is significantly up or down-regulated in cancer cells, and 2) they are co-expressed with a significantly higher number of enzyme-coding genes that have shared metabolic links. Thus, altering the expression of the key enzyme-coding genes will likely impact cancer cell metabolism. Meanwhile, altering the concentrations of the substrates or products of the metabolic reactions catalyzed by the key enzyme-coding genes will likely have similar effects. In fact, Met-express predicts the key enzyme-coding genes as potential cancer drug targets, and their substrates/products as potential drug compounds. In the current study, because the PUD targets are known, it is possible to combine the two types of predictions (KPC enzymes and PUDs) to select candidate drugs for treating pancreatic cancer. In addition, this approach offers the opportunity to hypothesize the molecular mechanism by which the selected candidate drugs may work against pancreatic cancer, either by targeting a KPC enzyme or by interfering with essential enzymatic reactions in cancer cells.

Although traditional screening approaches can identify PUDs, understanding their mechanisms remains a challenge. The absence of such information may delay the commercialization of PUDs, highlighting the advantage of using our combined approach. Additionally, as a general method, our combined approach can be applied readily to other types of cancer and other complex diseases, enhancing our ability to identify candidate drugs.

## Supporting Information

S1 Table The predicted key enzyme-coding genes for each of the three pancreatic cancer datasets. (XLSX)

S2 Table The enriched pathways and biological processes for the KPC enzymes predicted in all three cancer datasets. (XLSX)

S3 Table MTT assay results of pancreatic cancer cell lines. (XLSX)
